# The PPARg System in Major Depression: Pathophysiologic and Therapeutic Implications

**DOI:** 10.3390/ijms22179248

**Published:** 2021-08-26

**Authors:** Philip W. Gold

**Affiliations:** National Institutes of Health, Bethesda, MD 20892, USA; philipgold@mail.nih.gov; Tel.: +1-301-605-5902

**Keywords:** depression, PPARg, inflammation, neuropathology, corticotropin releasing hormone, norepinephrine, subgenual prefrontal cortex, amygdala, nucleus accumbens

## Abstract

To an exceptional degree, and through multiple mechanisms, the PPARg system rapidly senses cellular stress, and functions in the CNS in glial cells, neurons, and cerebrovascular endothelial cell in multiple anti-inflammatory and neuroprotective ways. We now know that depression is associated with neurodegeneration in the subgenual prefrontal cortex and hippocampus, decreased neuroplasticity, and defective neurogenesis. Brain-derived neurotrophic factor (BDNF) is markedly depleted in these areas, and is thought to contribute to the neurodegeneration of the subgenual prefrontal cortex and the hippocampus. The PPARg system strongly increases BDNF levels and activity in these brain areas. The PPARg system promotes both neuroplasticity and neurogenesis, both via effects on BDNF, and through other mechanisms. Ample evidence exists that these brain areas transduce many of the cardinal features of depression, directly or through their projections to sites such as the amygdala and nucleus accumbens. Behaviorally, these include feelings of worthlessness, anxiety, dread of the future, and significant reductions in the capacity to anticipate and experience pleasure. Physiologically, these include activation of the CRH and noradrenergic system in brain and the sympathetic nervous system and hypothalamic–pituitary–adrenal axis in the periphery. Patients with depression are also insulin-resistant. The PPARg system influences each of these behavioral and physiological in ways that would ameliorate the manifestations of depressive illness. In addition to the cognitive and behavioral manifestations of depression, depressive illness is associated with the premature onsets of coronary artery disease, stroke, diabetes, and osteoporosis. As a consequence, patients with depressive illness lose approximately seven years of life. Inflammation and insulin resistance are two of the predominant processes that set into motion these somatic manifestations. PPARg agonists significantly ameliorate both pathological processes. In summary, PPARg augmentation can impact positively on multiple significant pathological processes in depression. These include loss of brain tissue, defective neuroplasticity and neurogenesis, widespread inflammation in the central nervous system and periphery, and insulin resistance. Thus, PPARg agonists could potentially have significant antidepressant effects.

## 1. Introduction

The World Health Organization ranks depression as the second greatest cause of disability worldwide. In addition to its potentially crippling affective and cognitive elements, depression is associated with the premature onset of multiple systemic diseases, including coronary artery disease [1], stroke [2], diabetes [3,4], and osteoporosis [5]. These disorders are primarily mediated by the same changes in the central nervous system that transduce the cognitive and affective components of depressive illness. Overall, patients with depressive illness lose approximately seven years of their lives because of these somatic manifestations of the disorder [6].

Multiple lines of evidence indicate that depression represents a stress system that has become highly dysregulated [7,8,9,10,11]. Among the key regulators of the stress response in the brain and periphery is the peroxisome proliferator activated receptor gamma (PPARg) system, which I postulate plays a major role in depression, especially in one of the most pronounced concomitants of depression, namely central and peripheral inflammation [12]. Inflammation in these loci contributes to the cognitive and affective components, as well as to their somatic manifestations [9]. This paper will first document that the stress system runs awry in depression. I will also describe the organization of the stress system and the structural changes of the stress system during stress, provide a brief overview of the behavioral manifestations of depression, and detail the changes in structure and function that the stress system undergoes in encoding the clinical and biochemical manifestations of depressive illness. Finally, I will provide an overview of the biological changes associated with depression, with particular emphasis on the extensive roles of the PPARg system in depressive illness. This roles of stress system dysregulation in depression and of the PPARg’s role in regulating the stress system response and its dysregulation in depression are extremely important, and hence will be the highlights of this publication [13].

**Figure 1:**
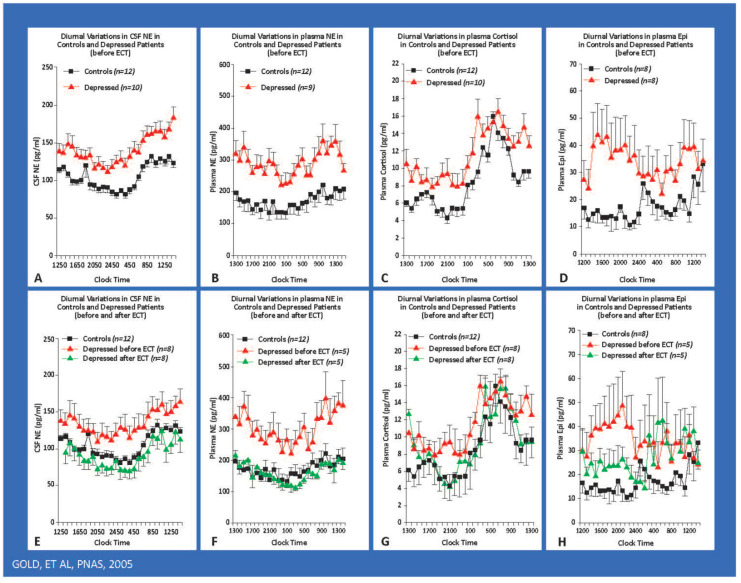
The hypernoradrenergic state of depression as assessed by around-the-clock hourly CSF and plasma sampling. The hypernoradrenergic state of melancholic depression. Hourly around-the-clock sampling of CSF NE, plasma NE, plasma cortisol, and plasma epinephrine in severely depressed, medication-free patients with melancholic depression. We studied patients during depression and after ECT-induced remission. Severely depressed patients had concomitant elevations of the hourly 24 h levels of CSF NE, plasma NE, plasma and CSF epinephrine, and plasma cortisol. These levels all fell to normal levels after ECT. The diurnal variations of CSF NE, plasma NE, and plasma cortisol were virtually superimposable and highly correlated with one another. Their arithmetic means also were highly correlated. These arousal-producing compound levels all peaked at 08:00 to 09:00, a time when melancholic symptoms are at their worst. Their peaks also coincide with the time for maximal susceptibility to myocardial infarction and sudden death. CSF NE and plasma norepinephrine correlate with one another, yet they derive from different sets of neurons. Patients with the Shy Drager syndrome have very low plasma norepinephrine levels in association with robust CSF norepinephrine concentrations. Excessive central norepinephrine secretion in melancholia exerts several adverse effects. Norepinephrine inhibits critical structures in the prefrontal cortex such as the subgenual and dorsolateral prefrontal cortices. NE stimulates the amygdala and the CRH/HPA axis. Central noradrenergic excess also contributes to hypertension, activation of the HPA axis, and the sympathetic nervous system. Plasma and CSF epinephrine were also elevated around the clock.

**Figure 2:**
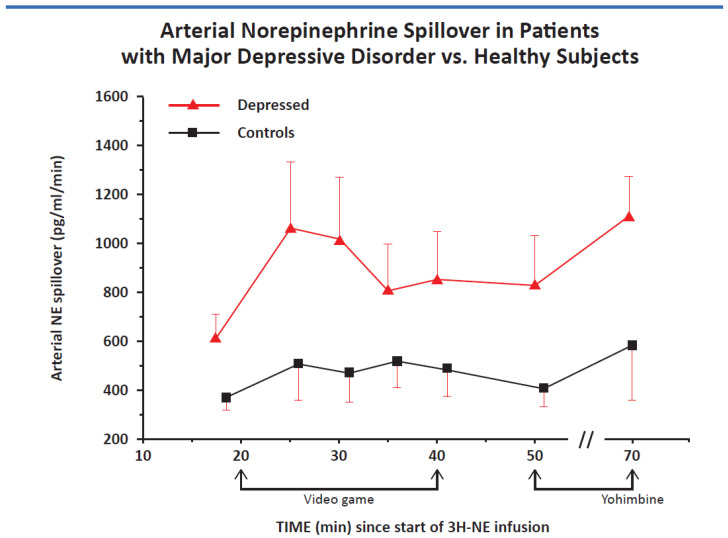
Norepinephrine spillover into arterial plasma corrected for norepinephrine clearance at baseline, during the stress of a video game, and after the infusion of yohimbine, an a-2 noradrenergic antagonist that increases the secretion of norepinephrine by blocking the inhibitory norepinephrine a-2 receptor (*p* < 0.01) for all findings.

There are two principal subtypes of depression that I will show have different manifestation of stress system dysfunctional activity. These are melancholic and atypical depression [9]. Melancholic depression is inconsistent with the word depression in that it is often a state of hyperarousal and anxiety, often attached to the self and experienced as an anguished sense of worthlessness compounded by insomnia, anorexia, and a host of metabolic and physiologic perturbations that interfere with the quality of life while significantly reducing the lifespan. Patients with melancholia have increased secretion of the stress hormones cortisol and norepinephrine. Their symptoms are worse in the morning when the stress system is at its peak. As an example of an activated stress system in depression, Figure 1 shows striking elevations in CSF and plasma norepinephrine and epinephrine levels in hourly samples drawn for thirty consecutive hours. Figure 2 shows increased norepinephrine spillover in mild–moderately depressed patients with melancholia.

Atypical depression seems to be the antithesis of melancholic depression and is associated with lethargy, fatigue, hypersomnia, and hyperphagia. The symptoms of atypical depression peak in the evening, when the stress system is most quiescent.

Rene Spitz made important observations regarding developmental abnormalities that afflicted infants placed soon after birth in understaffed orphanages, where they had little or no sustained human contact. Initially, the infants cried bitterly for hours until attended to. Subsequently, they withdrew and ceased crying at all, even if they were left alone or had gone without eating for hours. In addition, they lost interest in their environment. Thus, it were as if their early deprivation had led to a virtual shutdown of their stress response and affective existence to protect them from enormous distress [14]. Subsequent studies in non-human primates who were removed from their mothers and raised by peers reveal a similar behavioral withdrawal in association with significant inhibition of the HPA axis [15]. These may represent very severe forms of atypical depression.

Much less is known about atypical depression than about melancholia, thus much of this paper is focused on this depressive subtype. Overall, the weight of available data indicates that melancholic depression reflects a stress system that is pathologically activated, while atypical depression reflects a stress system that has been pathologically suppressed.

## 2. Organization of the Normal Stress System: Template for Depressive Illness

Anxiety is a cardinal manifestation of the stress response and is essential for survival. The PPARg system is highly activated during stress and plays multiple roles [16], including an anxiolytic one [17]. PPARg receptors are widely distributed in the amygdala and in the medial prefrontal cortex [18].

The CRH system is activated during stress. CRH is primarily in the hypothalamus and amygdala, and transduces activation of the hypothalamic–pituitary–adrenal axis, while amygdala CRH transduces anxiety and conditioned fear. Accordingly, CRH sets into motion multiple behavioral and physiological phenomena during stress. These include anxiety, hyperarousal, fear-related behaviors, activation of the sympathomedullary system, and hypothalamic–pituitary–adrenal activation. CRH is a potent stimulus to the activation of inflammation, which I shall show is highly increased during physical and psychological stress and in depression [9]. Inflammation is activated during stress as a premonitory response to the likely contingency of injury during stressful confrontations [9]. CRH diminishes sleep, food intake, sexual activity, and the capacity to anticipate or experience pleasure [9]. These actions serve many functions. One of their most important functions is to prevent distraction during threatening situations. The PPARg system restrains the CRH system and the activation of the sympathetic nervous system [19]. Thus, attention is directed primarily to the danger at hand.

As noted earlier, as examples of the activation of the stress response, we first noted that both CSF and plasma norepinephrine and epinephrine are elevated around the clock in drug-free patients with melancholia in hourly samples taken for 30 consecutive hours (Figure 1). Both compounds are arousal-producing and anxiogenic in brain and transduce multiple autonomic and metabolic aspects of the stress response [8,10,20]. We also found norepinephrine spillover was significantly increased in mild–moderately melancholic depressed patients [20] (Figure 2).

The dorsolateral prefrontal cortex is modestly inhibited, leading to a decrease in the cognitive control of anxiety [21]. Emotional memories of past confrontations with stress or danger are readily retrieved to support survival in the present threatening situation.

Stress is accompanied by a small, but significant down-regulation of the subgenual prefrontal cortex [9,22]. This structure restrains the amygdala fear system; estimates the likelihood of punishment and reward; helps prime the nucleus accumbens or reward center of the brain, thereby increasing the capacity to anticipate and experience pleasure. The subgenual prefrontal cortex also restrains the CRH and locus ceruleus-norepinephrine system and the sympathetic nervous system [9]. Taken together, this modest reduction in the size and functional activity of the subgenual prefrontal cortex leads to increased anxiety, increased expectation of harm, decreased capacity to anticipate or experience pleasure, activation of the locus ceruleus, and disinhibition of the CRH system and hypothalamic–pituitary–adrenal axis. As I will note, the PPARg system intersects with each of these important processes.

The amygdala, necessary for the cognitive experience of fear, is modestly disinhibited, partially through the decrease in the activity of the subgenual prefrontal cortex. Anxiety is kept manageable to prevent its interference with a successful stress response. The amygdala down-regulates the nucleus accumbens reward system to prevent distraction. It is difficult to experience pleasure when you are afraid. An activated amygdala also stimulates the CRH and sympathetic nervous systems via a CRH projection from the amygdala. As noted, the amygdala is amply supplied by PPARg receptors [17,18].

The hippocampus responds to cortisol and norepinephrine, which promote the encoding and retrieval of negatively charged emotional memories [23,24]. Mineralocorticoid receptors in the hippocampus exert negative feedback effects on the CRH system [25]. A direct projection from the anterior hippocampus to the subgenual prefrontal cortex modifies the subgenual prefrontal cortex during a stress response. The hippocampus also contains ample numbers of PPARg receptors [18].

The insula helps to control the shifting between the default mode network and the salient mode network. When the default mode system is activated in the context of an activated amygdala, as in depression, attention turns inward, leading to adverse self-assessments and contributing to the feelings of worthlessness, which are such critical elements in depression [17,26]. The default mode network is significantly hypoactive in depression and returns to its normal level of activation with successful antidepressant treatment. The insula has ample PPARg receptors that activate its capacity to promote a successful stress response [27].

As noted, the nucleus accumbens is modestly downregulated during stress, but not sufficiently to impede an effective stress response [28,29]. PPARg receptors are plentiful in the nucleus accumbens [18].

The locus ceruleus is the principal site of norepinephrine synthesis in the brain. It produces a state of general alarm, promotes anxiety, and plays an important role in activating the physiological aspects of the stress response.

During normal stress, there is a highly significant increase in neuroplasticity in the subgenual prefrontal cortex, amygdala, and hippocampus, and an increase in neurogenesis, all of which assist in effective response to stressful and rapidly changing circumstances [12]. Stress can also be associated with damage to brain cells that may be alleviated by PPARg agonists [12]. PPARg agonists are potent stimuli to both neuroplasticity and neurogenesis, which, I will show, are both markedly decreased in depression [12].

## 3. Behavioral and Cognitive Manifestations of Melancholic Depression

Melancholic depression is often associated with significant anxiety, hyperarousal, decreased appetite, and decreased sleep. As noted, the focus on the melancholic patient is on the inner self with active self-assessment, which, because of amygdala activation, is highly negative. Attention is focused on sad stimuli and there is significant difficulty in effectively disengaging from them. There is preferential access to negatively charged emotional memories that are highly resistant to extinction. Prior stressful events that are encoded in emotional memory and the affect associated with them are prominent in melancholia and are reinforced by cortisol and norepinephrine [9]. PPARg agonists down-regulate cortisol levels [12,30].

Recent data reveal that deletion of neuronal PPARg enhances the emotional response to acute stress and exacerbates anxiety. Importantly, these effects are reversed by rescue of amygdala PPARg function [17].

## 4. Dysregulation of the Stress System in Melancholic Depression: Evidence for a Pathological Activation of the Stress System

**Figure 3:**
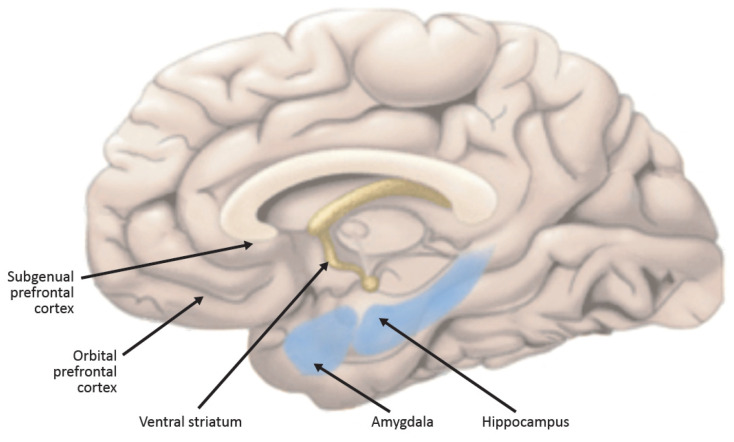
Sagittal section of the human brain. Structures playing a particularly important role in the pathophysiology of depression that are the targets of PPARg-amelioration of multiple core components of depressive illness. Please see text for descriptions, roles, and connections.

The size of the subgenual prefrontal cortex is reduced in melancholia by as much as 40% [31], owing to a loss of glial cells, with a marked decrease in its neuroplasticity, a decrease in the size of its neurons, and substantial loss of key synaptic proteins [32] (Figure 3). Stress and hypercortisolism are two factors that decrease the size and activity of this crucial structure [32]. As noted, one of the functions of the subgenual prefrontal cortex, an important component of the default mode network, is to participate in the process of self-assessment, and its impairment leads to a loss of self-esteem. Earlier, I pointed out that the subgenual prefrontal cortex is an important component of the medial prefrontal cortex, and although PPARg receptors have not been assessed in the subgenual prefrontal cortex, they are abundantly present in the medial prefrontal cortex [18].

As noted earlier, we first demonstrated that the CRH system was activated in melancholic patients [11], as were indices of increased norepinephrine secretion, as assessed hourly through indwelling canullae in the antecubital space and spinal canal (Figure 1). The highly elevated levels in melancholia fell to normal after electroconvulsive shock induced remission [8,10,20]. We also showed that melancholic depressed patients have increased norepinephrine spillover into arterial plasma at rest, in response to a video game, and in response to yohimbine, an alpha-2 noradrenergic antagonist (Figure 2) [20]. Along with CRH hypersecretion in melancholia, these lead to further anxiety and arousal, decreased sleep, and decreased food intake and sexual activity. Once again, chronic stress in experimental animals leads to these changes.

The nucleus accumbens (Figure 3), ordinarily primed by the subgenual prefrontal cortex, becomes enlarged and much less responsive to pleasurable stimuli. This leads to anhedonia, one of the cardinal manifestations of depression. Hypercortisolism contributes to this phenomenon [9]. The nucleus accumbens is amply supplied with PPARg receptors.

The hippocampus is decreased in size in depression (Figure 3). Neurogenesis is markedly reduced. Neuroplasticity is also significantly diminished. There is a decrement in BDNF that is a principal cause of these phenomena [33]. Ample data report that PPARg agonists significantly increase BDNF levels. The BDNF deficiency in depression is an important component of its pathophysiology. Some feel that it is among the most critical pathophysiological mediators in depressive illness [33,34]. Thus, PPARg agonists’ capacity to increase BDNF could be an important component of its possible therapeutic effects in depressive illness [35].

In addition, the dorsolateral prefrontal cortex is reduced in size in depression. Rich in PPARg receptors, the dorsolateral prefrontal cortex loses capacity to exert emotional control over cognition.

Depressive illness is not only associated with changes in structure and the function of sites altered in depression, but also with synaptic loss and deficits in functional connectivity [36,37,38,39]. Post-mortem research has demonstrated lower numbers of synapses and, correspondingly, lower expression of synaptic function-related genes in the dorsolateral prefrontal cortex in patients with depressive illness, consistent with the loss of lower levels of synaptic signaling proteins in these patients [40]. Lower synaptic density is associated with higher severity of depressive symptoms [40].

Almost all the manifestations of melancholia are corrected, either partially or fully, by effective antidepressant treatment [34,36,37,41,42,43,44,45]. These include partial restoration of the volume of the subgenual PFC and its functions [46], restoration of the hypoactivity of the dorsolateral prefrontal cortex [21,40], reduction in the size and activity of the amygdala [47], restoration of the volume of the hippocampus, normalization of its neuroplasticity, and restoration of neurogenesis [48]. There is normalization of the activations of the CRH and catecholaminergic systems, as well as of the growth hormone and reproductive axes [49,50]. BDNF levels rise significantly in the PFC and the hippocampus [33]. In addition, there is a decrease in the size of the nucleus accumbens and restoration of the normal capacity to experience pleasure. Cognitive function improves significantly [35,51]. Inflammation, which I will discuss extensively in the next section as a key component of depressive illness, also falls to normal levels [52].

## 5. Inflammation in Depression

Inflammation is an important component of depressive pathophysiology. Over 10,000 papers have been written on inflammation in depression. The PPARg system has highly significant effects in the restraint of multiple form of inflammation. For this reason, I will cover inflammation in depression in more detail.

Inflammation in Depression: A Key Factor that Contributes to Making PPARg Agonists of Relevance to Depression

Inflammation is an inherent component of depressive illness. This inflammation both influences the brain and is widespread in the rest of the body [53,54]. Thousands of papers have been written concerning the connection between inflammation and depression. Recent studies of animal models of stress such as social defeat, predatory stress, and resident intruders show that such stressors induce central nervous system inflammation, characterized by the secretion of cytokines, and evidence of neuronal inflammation [54]. This inflammation occurred in many areas of the brain thought to be involved in depression such as the subgenual prefrontal cortex and the amygdala [12,26]. The administration of blockers to inflammatory compounds blocked the impact of the stressors on behavior, including a depression-like picture. The administration of antidepressants prior to severe stress prevented any signs of neuroinflammation. Similarly, antidepressants in humans correct the evidence of peripheral and central inflammation [44,45,53].

Before discussing the many manifestations of inflammation in depressive illness, I would first like to discuss the evolutionary and biological roots in environments long ago that have fostered the close connection between depression and inflammation [53,54]. Almost all stressful situations that mammals encountered included risks implicit in being hunted, hunting, or competing for reproductive status or access. Thus, there was a premonitory activation of the inflammatory system anticipating possible injury and infection, because the risk of pathogen exposure and a consequent infection was highly increased [53,54]. Thus, in ancient environments, the connection between the perception of stress or danger and the risk of subsequent tissue injury was so expectable that evolution favored organisms that activated responses of inflammatory systems to a wide variety of environmental stressors, including psychosocial stressors [53,54]. Inflammatory mediators are among the most potent stimuli leading to the activation of prominent stress mediators such as activations of the sympathetic nervous system and hypothalamic–pituitary–adrenal axis. Data show that concentrations of IL-6 as low as 10^−18^ molar activated hypothalamic CRH neurons and the hypothalamic–pituitary–adrenal axis [9].

CRH plays a major role in neuron–microglial interactions in the CNS [47]. Among the most powerful microglia-activating factors is CRH, which plays many other roles in inflammation. CRH converts resting microglia into activated microglia, an effect suppressed by antidepressants [55], which we have shown to consistently down-regulate the CRH system [25,56]. Activated microglia lead to inflammation mediated by multiple cytokines and other proinflammatory compounds such as those associated with oxidative stress [55]. As noted, CRH is also among the most potent stimuli to mast cell degranulation in the CNS, the blood brain barrier, and the periphery [57,58,59]. Thus, antalarmin, our CRH antagonist, seems like an ideal candidate to address a primary mechanism in this CNS inflammation. As noted, PPARg agonists have consistent and multiple anti-inflammatory effects in the CNS, to be covered in more detail below.

Consistent, with the evolutionary advantages of the partnership between the brain and the immune system, inflammatory mediators in the brain, including CRH and cytokines, influence brain areas that regulate motivation, motor activity, areas promoting social avoidance and energy conservation, as well as arousal and anxiety, and fear, providing warning against attack [9]. Inflammatory mediators have also been associated with reduction in reward responsiveness, thus decreasing the adverse consequences of distraction by pleasurable stimuli such as sexual activity and food consumption [9].

Stress-mediated activation of the CRH system also leads to the release of CRH from sympathetic terminals in the periphery [50]. CRH in a potent inflammatory mediator, in the periphery. This provides an explanation for the mechanism of stress-induced skin disorders that occur because of stress, including urticaria.

Women have a greater behavioral response to endotoxin and a much higher depression rate in the context of gamma-interferon administration [53]. Thus, by being more responsive to inflammation-induced depressive symptoms, women may have benefitted more from the protection conferred by these symptoms in terms of coping with infection, healing wounds, and subsequent exposure to pathogens [54]. Inflammation also inhibits fertility. This may have protected women from unwanted pregnancy during times of adversity. It is well-known that depression occurs almost twice as frequently in women than in men. It should be noted that inflammation in the context of stress in both women and men is a sterile information [12], unrelated to direct exposures to pathogens or the induction of physical injury. The sterile nature of inflammatory stress may work through substances that, for instance, induce oxidative stress. Sterile inflammation is also very sensitive to catecholamines.

Another term for sterile inflammation is parainflammation. Among the manifestations of parainflammation is a low level, persistent activation of inflammation characterized by modest elevation in the levels of the inflammatory marker CRP, well-known to be a predictor of heart disease [12,26].

Parainflammation occurs in response to stressors such as overfeeding or aging that were not present during our early evolutionary history, and for which we are not adequately prepared. These also include alterations in the light/dark cycle and exposure to novel foodstuffs or chemicals [12]. I have suggested that the kind of frequent daily psychological stressors we encounter in our lives now was not also present in our early evolutionary history, and that stress-responsive illnesses like depression may be a parainflammatory disorder as well [12]. Parainflammation is likely to contribute to the chronic inflammatory conditions associated with modern human diseases, resulting from stimuli to which we were not exposed early in our evolution.

The difference between classical inflammation and parainflammation is that the latter does not occur in response to pathogens or tissue damage, but rather from alterations from the normal set-point in tissues in response to stressors such as those involving nutrient sensing, energy metabolism, oxidant burden, endocrine regulation, and autonomic stability [12]. Parainflammatory mediators have effects beyond those on inflammatory phenomena, and help coordinate endocrine, metabolic, and autonomic activity as well [50]. Chronic stress is likely to set into motion alterations in the normal set-point for endocrine, metabolic, and inflammatory processes that build to such a degree that, in individuals who are genetically susceptible, depression develops.

## 6. Manifestation of Inflammation in Patients with Depressive Illness

Inflammation in the body and the brain is a prominent component of depressive illness, to the point that multiple studies conclude that anti-inflammatory agents are useful in the treatment of depression [53,54]. Patients with major depression have been found to have increased peripheral blood inflammatory biomarkers, including inflammatory cytokines, compounds produced by immune cells that activate other immune cells to encode a significant inflammatory response. These peripheral cytokines have been shown to access the brain and interact with virtually every pathophysiologic domain known to be involved in depression. These include neurotransmitter metabolism, neuroendocrine function, neurogenesis, and neural plasticity, as well as inflammation [53,54]. Indeed, activation of inflammatory pathways within the brain is believed to contribute to a confluence of decreased birth of new neurons, decreased neuroplasticity, increased glutamate release, as well as oxidative stress, leading to destruction of neurons and loss of glial elements, cells that provide nutritional and other support to nerve cells.

Depressed patients with increased inflammatory biomarkers have been found to be more likely to exhibit treatment resistance [54]. Moreover, multiple studies in depressed patients have demonstrated that antidepressant therapy leads to decreased inflammatory responses. Finally, preliminary data from patients with inflammatory disorders, as well as medically healthy depressed patients, suggest that inhibiting proinflammatory cytokines or their signaling pathways may improve depressed mood and increase treatment response to conventional antidepressant medication [45]. These findings include the possibility of identifying relevant patient populations, applying immune-targeted therapies, and monitoring therapeutic efficacy at the level of the immune system in addition to behavior.

Stressed experimental animals have central nervous system inflammation, characterized by the secretion of cytokines and evidence of neuronal inflammation. This may occur, in part, because of increased glutamate activity in depression [39]. Glutamate is a potent stimulus to central nervous system inflammation. This inflammation occurs in many areas of the brain thought to be involved in depression such as the subgenual prefrontal cortex and the amygdala.

## 7. More on the PPARg System

The PPARg receptor is a nuclear receptor found in neurons, glia, and cerebrovascular vessels in the frontal cortex, nucleus accumbens, striatum, hippocampus, hypothalamus, and substantia nigra. To an exceptional degree, and through multiple mechanisms, the PPARg system in the brain is widespread and rapidly senses CNS cellular stress [57] and functions in the CNS as a potent anti-inflammatory agent that protects neurons, glial cells, and cerebrovascular endothelial cells [58,59,60,61,62,63,64,65] from many inflammatory mediators of the innate immune system such as NF-kB, TNF-a, IFNg, IL-1b, and IL-6, as well as COX2, MCP1, and VCAM1.

In addition to these pleiotropic anti-inflammatory properties, the PPARg system is neuroprotective and affords protection from oxidative stress [60] and inappropriate apoptosis owing to excessive endoplasmic reticulum stress [61,62] and other stimuli. Thus, interference with the central PPARg system is associated with a pronounced down regulation in the availability of SOD-1 and glutathione S transferase [63], klotho, which functions as an anti-oxidant [64,65,66,67], and a marked increase in susceptibility to the adverse effects of excessive NMDA neurotransmission [19,68,69]. Interference with chaperones of the endoplasmic reticulum stress response, which promote a successful response, impairs the capacity for the endoplasmic reticulum stress response to handle oxidative stress and other neuronal stressors [70]. The PPARg system also promotes neuroplasticity and neurogenesis during periods of neuronal stress [71].

In addition to directly mediating anti-inflammatory effects and neuroprotection directly, the PPARg system is essential to the anti-inflammatory effects of compounds like angiotensin receptor-1 antagonists [72], whose anti-inflammatory responses are completed abolished by antagonism of neuronal PPARg receptors [72].

PPARg agonists also inhibit the CRH system [19], as well as the neurotoxic effects of norepinephrine [73] and glucocorticoid excess [19]. The striking anti-neuroinflammatory and neuroprotective effects of PPARg agonists have led to the recent initiation of pioglitazone, a PPARg agonist that crosses the blood brain barrier, to either treat or delay the progression of a variety of neurodegenerative diseases including Alzheimer’s disease [74,75], Parkinson’s disease [75], Huntington’s disease [75], Friedrichs’ ataxia [75], and the demyelination of multiple sclerosis [75]. In the periphery, an intact PPARg system is essential for ketamine-induced suppression of the innate immune system.

Prior to the elucidation of PPARg-mediated anti-inflammatory and cellular protective mechanisms in both the periphery and the CNS, the therapeutic efficacy of PPARg agonists was thought to reside solely in its capacity to significantly increase peripheral insulin sensitivity, protect pancreatic beta cells, and indirectly provide cardioprotection by ameliorating insulin resistance [76]. Thus, initially, PPARg agonists have been used primarily in the treatment type II diabetes, which is almost always associated with atherosclerosis and widespread inflammation [77]. Thus, because insulin is a potent stimulus to inflammation, pioglitazone also exerts a marked anti-inflammatory response in the periphery and brain.

It is now firmly established that insulin in the CNS plays a pronounced role in promoting adaptive neuroplasticity and in protecting from oxidative and glutaminergic stress via the widely distributed insulin receptor substrate p53, which plays a key role in modulating the actin cytoskeleton and the remodeling of dendritic extensions [78,79,80]. Insulin in the brain derives solely from the periphery via active transport across the blood brain barrier. In states of insulin resistance and peripheral hyperinsulinemia, insulin transport is reduced because of the saturation of insulin receptors in the blood brain barrier [81]. Thus, peripheral insulin resistance, which we see in our depressed patients, is likely to be associated with a CNS insulin deficiency and associated with disturbed neuroplasticity, increased susceptibility to oxidative and glutaminergic stress, and inflammation.

We now know that overfeeding in mice results in a primary neuroinflammation, associated with activation of the intraneuronal NF-kB system [82]. This form of autonomous intraneuronally-mediated inflammation is unique, in contrast to canonical neuroinflammation that is activated via proinflammatory mediators deriving from glial immunocompetent cells (the canonical form of neuroinflammation) [82]. Blockade of this response in hypothalamic neurons by NF-kB antagonists eliminates the effect of overfeeding on peripheral insulin resistance, thus establishing the brain as the primary initiation site in the kind of insulin resistance we see in patients with major depression [82].

This autonomous intraneuronal inflammation has been designated as parainflammation [12]. Parainflammatory responses are unique in that they occur in the context of stressors for which we were evolutionarily unprepared, including not only overfeeding, but also marked decreases in physical activity, aging, disturbances due to loss of exposure to the naturalistic light–dark cycle and sleep deprivation, as well as novel foods and drugs [12]. We postulate that repeated acute social stressors that may not have been present during our early evolution may also set into motion parainflammatory responses related to increased NF-kB activation. Insulin and PPARg receptors in the CNS are among the most inhibitory modulators of neuronal NF-kb activity [12].

A key marker for parainflammation in the periphery is a smoldering, subtle 50–75% elevation in the level of acute phase proteins such as CRP, in contrast to CRP responses to infection that rise quickly 100-fold or more. Smoldering CRP elevations are seen in coronary artery disease, which was likely to be rare in our early history, as well as in states of major depression.

We have found that, compared with unmedicated, remitted patients with major depression, remitted patients receiving specific serotonin uptake inhibitors (SSRI) treatment are insulin-resistant, hyperinsulinemic, and have significantly higher levels of plasma glucose (preliminary observations). Two well-controlled epidemiological studies have shown that patients on SSRI treatment have a 2–3-fold increase in the incidence of type II diabetes. These data indicate that there might be a dissociation between the positive impact of SSRIs on the affective and cognitive components of the depressive syndrome from the systemic stigmata that were assumed to occur only during the depressed state.

PPARg agonists have activity on a striking multiplicity of interrelated pathophysiological CNS processes we now know occur in patients with major depression. Therefore, we propose a placebo-controlled, double-blind trial of pioglitazone, a safe and potent PPARg antagonist that crosses the blood brain barrier. Pioglitazone’s CNS anti-inflammatory, neuroprotective, and neurotropic effects intersect with virtually every known pathophysiologic parameter identified in patients with major depression. Moreover, its insulin-sensitizing effects in the brain would complement these actions, and the amelioration of insulin resistance in the periphery would not only correct plasma hyperinsulinemia and decreased availability of insulin in the CSF, but also the highly pathogenic sequalae of insulin resistance on the quality of health and the lifespan. Data in experimental animals reveal that PPARg agonists exert behavioral effects interpreted as antidepressive. One open trial without placebo control [83] reported that pioglitazone has antidepressant properties in depressed patients.

**Figure 4:**
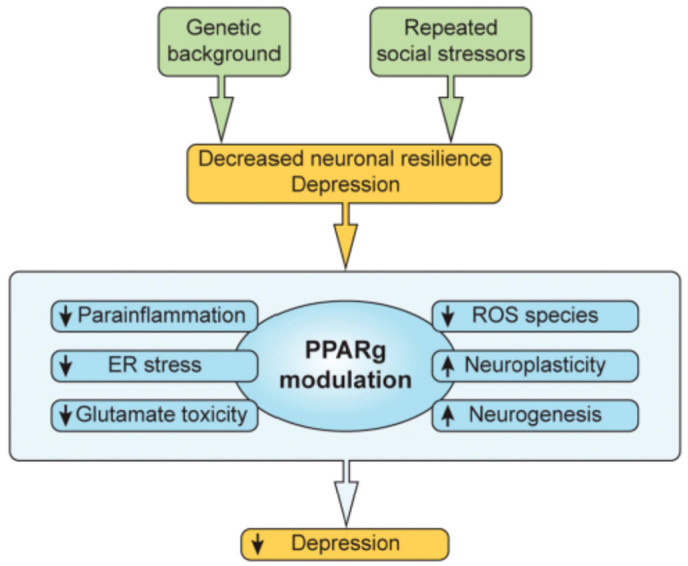
Repeated social and other stressors plus genetic predisposition lead to decreased neuronal resilience that can, in turn, lead to depression. Processes set into motion include parainflammation, extreme endoplasmic reticulum stress responses, glutamate toxicity, BDNF function, and the regulation of central and peripheral insulin dynamics. The PPARg system can modulate and diminish each of these pathologic drivers, and others as well, as noted in the text.

In summary, PPARg augmentation can impact multiple significant pathophysiological inflammatory, neurotransmitter, and neuroendocrine processes involving peripheral and central insulin regulation, as well as intracellular processes critical to transducing the clinical and biological manifestations of depressive illness. These include processes such as parainflammation, multiple inflammatory pathways in the brain and periphery, endoplasmic reticulum stress, neuroplasticity, neurogenesis, BDNF-mediated processes, neutralization of oxidative stress, the sequela of glutamate toxicity, and the consequences of hypercortisolism (Figure 4).
